# Exploring the implementation of patient-reported outcome measures in cancer care: need for more real-world evidence results in the peer reviewed literature

**DOI:** 10.1186/s41687-018-0091-0

**Published:** 2018-12-27

**Authors:** Milena Anatchkova, Sarah M. Donelson, Anne M. Skalicky, Colleen A. McHorney, Dayo Jagun, Jennifer Whiteley

**Affiliations:** 1 Patient-Centered Research, Evidera, Bethesda, MD USA; 2 Genentech Inc., South San Francisco, CA USA; 3 San Francisco, California USA; 4 Tampa, Florida USA

**Keywords:** Patient-reported outcomes, Clinical practice, Quality of care, PRO implementation, Patient-centered care, Cancer, Oncology

## Abstract

**Background:**

To explore the existing evidence of the real-world implementation of patient-reported outcomes (PROs) in oncology clinical practice and address two aims: (1) summarize available evidence of PRO use in clinical practice using a framework based on the International Society for Quality of Life Research (ISOQOL) PRO Implementation Guide; and (2) describe reports of real-world, standardized PRO administration in oncology conducted outside of scope of a research study.

**Methods:**

A Preferred Reporting Items for Systematic Reviews and Meta-Analyses (PRISMA) protocol was developed to guide the systematic literature review (SLR) that was conducted in MEDLINE and Embase databases. A two step search strategy was implemented including two searches based on previously completed reviews. Studies published from 2006 to 2017 were synthesized using a framework based on the ISOQOL PRO Implementation Guide.

**Results:**

After screening 4427 abstracts, 36 studies met the eligibility criteria. Most elements of the ISOQOL PRO Implementation Guide were followed. Two notable exceptions were found: 1) providing PRO score interpretation guidelines (39% of studies); and 2) providing patient-management guidance for addressing issues identified by PROs (25% of studies). Of the 22 studies with an intervention component, 19 (86%) reported intervention effects on study outcomes. The European Organisation for Research and Treatment of Cancer Quality-of-Life Questionnaire-Core 30 (EORTC QLQ-C30) was the most commonly used PRO (*n* = 10, 28%); use of 38 other PRO measures was also reported. Only three studies (8%) reported real-world PRO implementation.

**Conclusion:**

Reports of real-world PRO implementation are limited. Reports from studies conducted in clinical settings suggest gaps in information on PRO score interpretation and the use of PRO results to inform patient management. Before the promise of practice-based PRO assessment in oncology can be truly realized, investigators need to advance the state-of-the-art of real-time PRO score interpretation as well as developing guidance on how to use PRO insights to drive clinically-meaningful patient-management strategies.

## Introduction

There has been growing interest in the assessment of patient-reported outcomes (PROs) over the last 40 years, and the use of PROs in clinical and health services research is common [1]. PROs have been defined as “any report of the status of a patient’s health condition that comes directly from the patient, without interpretation of the patient’s response by a clinician or anyone else.” [2] The incorporation of PROs in clinical practice can serve numerous purposes [3] including: (1) describing a patient’s overall state; (2) screening for incipient disease and undetected disability [4, 5]; (3) monitoring disease progression and response to treatment; (4) assessing patient-centered needs; (5) formulating treatment plans consistent with patient preferences [6–8]; (6) improving physician-patient communication [9–11]; (7) providing patient-based data for quality initiatives [12–14]; and (8) standardizing interactions between healthcare providers and patients [3]. While the use of PROs in clinical practice can help in all of these areas [1, 15], critical questions remain about how patient outcome data should be collected, shared, and used to improve the quality of care and patient health outcomes [16]. Some reports have emerged regarding the use of PROs in routine clinical practice across different conditions [1, 17–22], but the incorporation of these tools in oncology clinical practice has been slower than adoption in research [23–26].

More recently, routine use of PROs in oncology practice has been identified as a priority area by the President’s Cancer Panel [27] as well as by national oncology societies such as the American Society of Clinical Oncology (ASCO) [28]. There is increasing interest in bringing “the patient’s perspective” to cancer decision making which is demonstrated by a number of key initiatives of PRO application in oncology research and regulatory decisions [29]. However, little evidence has been generated with regards to clinical-practice implementation [30].

The interest in implementation of PROs specifically in oncology care is exemplified by the number of recent reviews on PRO clinical applications and their impact on health outcomes [31–34]. All of the recent oncology reviews provide some insights on gaps in existing evidence of PRO use in clinical practice related to both challenges in implementation and PRO use impact. For example, Howell identified that more attention needs to be paid to complexity of implementation and interpretation [32]. King and colleagues [33] found a scarcity of studies reporting data on actions and medical decisions [33]. Two others—Chen [31] and Kotronoulas [34]—examined PRO intervention evidence and identified weak signals specific to changes in patient management and improved health outcomes [31, 34]. While these reviews identified important evidence gaps, none of them used an existing implementation framework to organize findings or focused on a review of publications reporting on the actual implementation of PRO in real-world settings beyond the context of a feasibility study or intervention trial. The current review makes a unique contribution to the field, by summarizing currently existing evidence using an implementation framework based on the user’s guide for the implementation of PROs in clinical practice recently developed by the International Society for Quality of Life Research (ISOQOL) [35]. The guide includes recommendations for the following implementation elements: (1) identifying the goals for collecting PROs in clinical practice and which key patient outcomes or barriers need attention; (2) considering group of patients and the care settings; (3) determining which questionnaire(s) to use (e.g., whether to use generic or disease-specific questionnaires, profile or preference-based measures, single or multi-item scales, and static or dynamic questionnaires); (4) choosing how often a patient should complete the questionnaires and whether it should be one-time completion or repeated, tied to clinic visits, or a way to monitor patients between visits; (5) deciding how the PRO will be administered and scored; (6) identifying interpretation benchmarks for the PRO score and how scores requiring follow-up will be determined; (7) developing strategies for when the PRO results will be presented and discussed with the patient (such as during or after the visit), how the results will be presented (e.g., numeric, graphical, one-time results or trends over time), and who will see the PRO score reports; (8) determining what will be done to respond to issues identified by the PROs and follow-up; and (9) evaluating the impact and value of the PRO interventions on the practice and patient [35]. While previous publications have discussed various considerations and potential applications of PROs in clinical practice [36–38], the ISOQOL PRO Implementation Guide is most recent and provides specific implementation guidance developed by subject matter experts and endorsed by a professional organization.

The objective of this systematic literature review (SLR) was to explore and summarize the existing evidence of PRO use in oncology clinical practice. We address two key aims in the review: (1) summarize available evidence of PRO use in clinical practice using a framework based on the ISOQOL PRO Implementation Guide [35]; (2) describe reports of real-world implementation of PRO measures with oncology patients. Real-world implementation of PROs can provide evidence regarding the usage and potential benefits of PRO adoption derived from real-world clinical settings. For the purposes of this review, real-world implementation studies were defined as those reporting the process of ongoing standardized PRO administration and related clinical actions to manage patient care conducted in a routine clinical practice beyond the scope of a specific research study.

## Methods

### Study design

We conducted a SLR in accordance with the Preferred Reporting Items for Systematic Reviews and Meta-Analyses (PRISMA) guidelines [39, 40]. The protocol was developed following PRISMA guidelines and the Guidance on the Conduct of Narrative Synthesis in Systematic Reviews [41].

### Data sources

The literature search was conducted in two databases: MEDLINE and Embase. Blocks of medical subject heading (MeSH) terms were used to identify the most relevant articles and conference papers that describe PRO implementation in oncology clinical practice.

### Search strategy

The SLR search strategy was developed in consultation with a professional librarian and used a two-step approach for identifying studies. The first step included a search strategy and approach based on a previously published systematic review of use of PROs in oncology care [31]. As part of the first step, two additional, closely-related systematic reviews were identified [32, 33]. References from these oncology literature reviews were examined, and studies meeting the selection criteria were incorporated into the review [32, 33]. As time has elapsed since the publication of these reviews, they had a narrower focus and used different search strategies and search terms, a second search was conducted to replicate and update the earlier Howell [32] and King [33] reviews to further ensure a comprehensive review of recent publications from the end date of published reviews. MeSH terms and free-text keyword groups (e.g., “neoplasm,” “PRO measure,” “clinical practice,” and “treatment”) were used in different combinations. These updated searches also included specific PROs as search terms, minimizing the risk of missing relevant articles that used these measure, but may have resulted in overrepresentation of these specific measures in the final results. Terminology adjustments were made according to the requirements of each database. Both searches were supplemented by a hand search of references of relevant articles. Appendix A shows the full search strategy.

### Selection criteria

Articles were included in the review if they met the following inclusion criteria: (1) cancer focus; (2) articles published in the past 10 years from 2006 to 2016 (inclusive) and abstracts from meetings held in 2015–2016 to ensure review of the current state of the field; (3) published in English; (4) title, abstract, or article contained information pertaining to the measurement of treatment satisfaction, process of care, treatment adherence, treatment decision-making, patient activation, PROs of health-related quality of life (HRQoL), symptoms, or function; and (5) the study design was a randomized controlled trial or an observational study in a clinical-practice setting or a report of PRO implementation in clinical practice.

Exclusion criteria were: (1) articles focused on non-cancer populations; (2) measures did not pertain to clinical outcomes or PROs associated with cancer treatment; (3) basic science studies (e.g., molecular biomarkers, neuroimaging drug formulation); (4) study designs not relevant including study protocols, case studies, case reports, case series, editorials, reviews, commentary, news, or study protocols; and (5) non-English language.

### Data screening and abstraction

All abstracts were reviewed using DistillerSR® [42]—a systematic literature review reporting software—to assist with the organization, extraction, and categorization of all literature. Abstract and article screening was performed by three trained reviewers in a two-step process. During the Level 1 review, in order to standardize the review process, a calibration exercise was conducted by the reviewers for all abstracts and titles to assess eligibility for inclusion in the full-text review. Full-text articles that met the inclusion criteria were retrieved. If a determination of eligibility was not possible from the abstract, the full-text article was reviewed. During Level 2, full-text articles were reviewed again for eligibility. Disagreements on eligibility of screened publications at both levels were resolved through discussion with reviewers and final adjudication of unresolved disagreements by the first author of this paper (M.A). For eligible articles, the data was abstracted into a detailed source table that included data fields on study country, study type, cancer type, study objectives, sample size, study duration, study inclusion/exclusion criteria, PRO intervention characteristics, PRO reporting characteristics, study endpoints, assessment timepoints, PRO study results, and limitations/contextualization. The data abstracted into the detailed source table was validated by a second independent senior reviewer to ensure the accuracy of data abstraction. The detailed source table was used to organize information in summary tables that were developed during data analysis and based on the ISOQOL PRO Implementation Guide Framework.

### Data analysis

We used the ISOQOL PRO Implementation Guide [35] as the basis for developing a framework to address our first research aim (Table 1). Categories corresponding to each of the ISOQOL PRO Implementation Guide recommendations were created, and information from the articles was extracted into summary tables from the original detailed literature source table. This was done to explore relationships in the data and to establish if all recommended information was included in reports of PRO use in oncology clinical practice. The framework was used to explore the use of PROs in clinical care and their relationship to outcomes in the context of the ISOQOL PRO Implementation Guide. To inform the second aim of this review—to describe reports of real-world implementation of PRO measures settings with oncology patients—we examined the characteristics of all real-world implementation reports.

**Table 1:** ISOQOL PRO Implementation Guide Framework for SLR Data Synthesis

	Data Synthesis Categories	Data Codes
PRO Study Design Recommendations	Goals for Collecting PRO	Screening, Monitoring, Patient Centered Care, Decision Aid, Team communication, Quality of care
	Patients, Setting, Timing of Assessment	Patients: Type of Cancer, Adults vs. Children
		Setting: Clinic, Home, Hospital, Hospice
		Timing of assessment: Before Visit, During Visit, After Visit
	Selection of PROs	Types of PROs Used: Symptoms, Function, Disease-Specific Quality of Life, Generic Quality of Life, Other
	PRO Mode of administration	Paper and Pencil /Phone/ IVR/ePRO (Tablet, Web, Phone)
PRO Study Results Recommendations	Reporting of PRO Results	Where? Clinical Flow vs. Other
		How? Numbers, Graphs, Full Report
		Who? Clinical Team, Patient, Both
	Score Interpretation	Written guidelines, Cut Scores, Minimally Important Difference, Normative Scores
	Plans for Addressing Issues Identified by PRO	Plans in Place/No Plans
	Evaluation of PRO Impact on Clinical Practice	Research Designs Used (RCT, Quasi RCT, Survey)
		Types of Outcomes Considered: Outcomes with Evidence of Impact

*Abbreviations*: *ePRO* = electronic patient-reported outcome, *IVR* = Interactive Voice Response, *PRO* = patient-reported outcome, *RCT* = randomized controlled trial

## Results

**Fig. 1:**
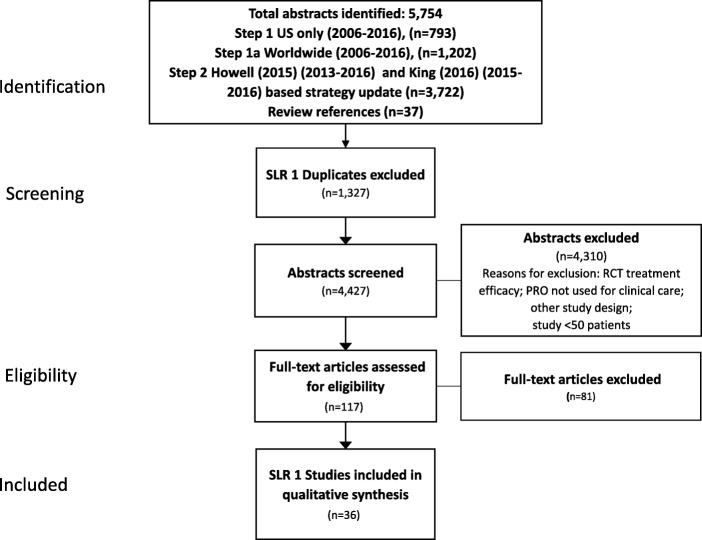
PRISMA Flow Chart

As shown in the PRISMA diagram (Fig. 1), a total of 5754 records were identified, and they yielded 4427 unique publications (following removal of duplicates). A total of 117 full-text articles were retrieved for a second round of screening. Of these, 36 articles met the inclusion criteria and are presented herein (Table 2). The largest number of studies were from the United States (US) (*n* = 16, 44%) followed by the United Kingdom (UK) (*n* = 6, 17%), Germany (*n* = 3, 8%), Canada (*n* = 2, 6%), The Netherlands (n = 2, 6%), other individual European Union (EU) countries (n = 6, 17%), and Australia (*n* = 1, 3%).

**Table 2:** Summary Table with Key Elements of Included Studies

Citation (author, year, title)	Patient/ Type of Cancer	Research Design	Study Goal	PRO Used	Member of medical team receiving feedback	PRO score interpretation	Plans for addressing issues identified by PRO	Study Results
Álvarez-Maestroa, M. 2014 [60]	Adults with metastatic prostate cancer	Cohort study	Feasibility	PROSQoLI, Perceived health status	Physician	Score information provided	No instructions given, only usual best practice guidelines	• % Physicians finding useful:
								o 66.1% clinical decision making
								o 71.3% questionnaire characteristics
								o 73.4% doctor---patient communication.
Basch, E. 2016 [61]	Adults with any cancer type	RCT	Intervention	EQ-5D and CTCAE	Oncologist, nurse	Score information provided	No instructions given, only usual best practice guidelines	• EQ-5D (*p* < .001)
								• ER Visits (*p* < .02)
								• 1 year survival (*p* = .05)^1^
								• # of Nursing calls (*p* = .93)
Basch, E. et al. 2007 [62]	Adults with any cancer type	Cohort study	Feasibility/ Intervention	EQ-5D and CTCAE	Clinicians	Score information provided	Standard AE reporting procedures	• 85% of participants logged in during clinical visits
								• 66% logged in from home
								• 57 Grade 3 or 4 Toxicity reported
Berry, D. 2014 [63]	Adults with any cancer type	Cluster RCT	Intervention	ESRA-C and SDS	Clinicians	Score information provided	Questions for patient to ask clinician	• ESRA Intervention group had a significant reduction (*p* < .05)
Berry, D. 2015 [64]	Adults with any cancer type	RCT	Intervention	ESRA-C and SDS	Patient	Graphical data display	Questions for patient to ask clinician	• Significant reductions of symptom distress in ESRA-C intervention group (*p* < .01)
Berry, L., et al., 2011 [65]	Adults with any cancer type	RCT	Intervention	ESRA-C and SDS	Clinicians	Score information provided	No instructions given, only usual best practice guidelines	• Significant interaction effect for reporting of symptom/QoL issues (*p* = .032).
								• More discussion in intervention group if SQoLs reported as problems.
								• 50–60% of clinicians found PRO report useful
								• No difference in study length
Blum, D. 2014 [66]	Adults with advanced cancer	Cohort study	Feasibility	E-MOSAIC	Physician	Graphical data display	Discussion of PRO results	• % Clinicians agreeing:
								• 77% Useful monitoring system
								• 38–95% Better symptom control
Boyes, A., et al. 2006 [67]	Adults with any cancer type	RCT	Intervention	Symptoms, HADS, Care needs	Oncologist	Score information provided	No instructions given, only usual best practice guidelines	• No significant differences between groups in HADS outcomes
								• Majority of patients founds the survey easy to complete, good way to communicate with doctors, willing to complete again.
Chiang., A. 2015 [68]	Adults with any cancer type	Cohort study	Intervention for quality Improvement	NCCN-EDT	Clinical team	None	Referral to social worker	• EMR documentation increased from 19% to 34% over 6mo before and after intervention
								• Barriers:
								• Insufficient time with patients, lack of social work resources, lack of privacy and space to discuss, and patient discomfort in discussing.
Compaci, G., 2015 [69]	Adults with lymphoma	Cohort study	Feasibility	HADS, PTSD-CL, SF-36, eCRF	Oncologist, nurse or GP	Score information provided	Phone follow-up, clinic consultations	• Time for whole intervention 55 min per quarter
								• Anxiety decrease 20% to 14% baseline to 12 months
								• Depression decrease 10% to 6.5% baseline to 12 months
								• PTSD 14.8% to 17.6%
Cox, A., et al., 2011 [70]	Adults with lung cancer	Qualitative interviews and Cross-sectional study	Feasibility	ESAS and EQ-5D	Clinicians	Score information provided	Questions for patient to ask clinician	• Clinicians found PRO beneficial, but only considered them complementary.
Engelen, V., et al., 2012 [71]	Children with any cancer type	Cohort study	Intervention	QLIC-ON, PEDSQL, TAPQoL	Oncologist	Not reported	Discussion of PRO results	• HRQoL domains discussed more in intervention group (p < .05)
								• Significantly more emotional and cognitive problems were identified in the intervention group compared to the control group.
								• Better HRQoL outcomes in intervention group for children 5–7 old, but not other age groups.
Epstein, R., 2017 [72]	Adults with any cancer type	Cluster RCT	Intervention	McGill QoL scale single item, McGill Psychological Well-Being subscale, McGill Existential Well-Being subscale, FACT-G Physical Functioning subscale, and FACT-G Social Functioning subscale.	Clinicians	None	Coaching session, follow-up phone calls, list of follow-up questions	• Significant intervention effect for the composite of patient centered communication measure reported (p < .02)
Erharter, A., et al., 2010 [73]	Adults with primary brain tumor	Cohort study	Feasibility	EORTC-QLQ-C30, EORTC-BN20	Physician	Score information provided	No instructions given, only usual best practice guidelines	• Time for completion decreased from 10 to 5 min over the course of the study.
								• Majority of patients and physicians found the PRO acceptable.
Hilarius, D., et al., 2008 [74]	Adults with any cancer type	Cohort study	Intevention	EORTC QLQ-C30, EORTC QLQ-CR38, EORTC QLQ-LC13, EORTC QLQ-BR23	Nurse	Score information provided	No instructions given, only usual best practice guidelines	• HRQoL-related topics discussed significantly more often in the intervention group (*p* = .009)
								• Awareness of HRQoL issues significantly better in intervention group at visit 4 (p = .05)
								• Significantly more HRQoL chart notations in intervention group (p < .001)
								• No significant differences in patient satisfaction
								• No significant differences in patient HRQoL at visit 4.
								• Evaluation of
								• Intervention: All nurses reported: the summary provided useful information, facilitated communication, resulted in a more efficient use of their time with the patients, a desire to continue using the HRQoL summary profiles in their daily practice.
								• Patients
								o 89% reported the
								HRQoL summary profile provided an accurate picture of their HRQoL, 69% reported it was used explicitly during treatment 89% believed that the summary enhanced their nurses’ awareness of their health problems, and 99% believed that it would be useful to introduce the intervention as a standard part of the outpatient clinic procedure.
Izard, J. 2014 [75]	Adults with prostate cancer	Cohort study	Feasibility	REALM-SD, SNS, Graphic Literacy Scale	Researcher	Graphical data display	Not reported	• Pictograph was the least preferred format
								• Patients favored the bar chart (mean rank, 1.8 [*P* = .12] vs line graph [P < .01] vs table and pictograph);
								• Providers favored bar (34%), and line (34% and Table (30%) formats.
Kallen, M., et a., 2012 [76]	Adults with any cancer type	Qualitative interviews and Cross-sectional study	Feasibility	ELVIS and ESAS	Providers, patients and caregivers	Graphical data display	Not reported	• Provider interview themes: Improved communication, Barriers to implementation
								• Patient interview themes: improved comprehension, improved communication, and improved patient peace-of-mind
								• Usability: physicians, nurses, patients, and caregivers endorsed the usability of the system (SUS score 83.9)
Mooney, K. 2014 [77]	Adults with any cancer type	RCT	Intervention	Symptoms	Oncologist, nurse, preferred provider	Cut-offs/Thresholds	Structured interview	• There were a total of 6509 calls into the system by 223 patients. The overall daily call adherence was 65.0% of expected days.
								• No significant intervention effect
Nicklasson, M., et al., 2013 [78]	Adults with lung cancer or mesothelioma	RCT	Intervention	EORTC QLQ-C30, EORTC LC13	Physician	Interpretation of PRO score reports	No instructions given, only usual best practice guidelines	• Only emotional functioning was more frequently discussed in the intervention group both by doctors (*p* = 0.018) and by doctors or patients taken together (*p* = 0.015).
								• More interventions aimed at emotional and social issues and dyspnea in intervention group (p < .05
Rogers, S.N. 2016 [79]	Adults with any cancer type	Cohort study	Intervention	UWQoLv4, PCI	Physician	Not reported	Not reported	• Median number of concerns 3 (range 1–6)
								• Significant association with number of dysfunction scores (*p* < .005)
Rosenbloom, S., 2007 [80]	Adults with advanced breast, lung or colorectal cancer	RCT	Intervention	FACT-G, FLIC, PSQ-III, POMS-17	Nurse	Score information provided	Structured interview	• No significant differences between groups on study outcomes
Ruland, C., 2010 [81]	Adults with acute myelogenous leukemia (AML), lymphatic leukemia (ALL), multiple myeloma, Hodgkin disease, or non-Hodgkin lymphoma,	RCT	Intervention	SF-36, CES-D, MOS-SS	Patient, clinicians	Patient importance or bothersome ranking	No instructions given, only usual best practice guidelines	• Significantly more symptoms and problems address in intervention group (p < .0001)
								• Positive intervention
								• Effect for discomfort, eating/drinking, sleep/rest, and sexuality.
								• Significant reduction in 10 of 19 symptom distress categories (p < .01)
								• Symptom management: Group differences statistically significant in favor of the intervention group in 13 of 19 (68%) categories.
Schuler, M. 2016 [82]	Adults with any cancer type	Cohort study	Feasibility	PRO-Onc, EQ-5D	Clinic staff	Not reported	Not reported	• Only 4 patients refused to participate
								• 68% staff reported PRO easy to handle
								• 22% used information for care planning during hospital stay; 52% rated additional time as minimal
								• HRQoL scores from admission to discharge (no control group)
Seow, H., et al., 2012 [43]	Adults with lung and Breast Cancer	Retrospective chart reviews	Implementation	ESAS	Oncologist and nurse	Clinically significant severity levels	Not reported	• ESAS score associated with increase in both documentation and actions for pain and shortness of breath
Siekkinen, M. 2015 [83]	Adults with breast cancer	Single-blinded, RCT	Intervention	FACT-Breast Cancer, STAI	Oncologist, nurse	Not reported	Patient education materials	• As ESAS severity score category increase associated with
								• Significant increase in proportion of visits with symptom documentation (pain and shortness of breath) (p < .0001)
								• Increase proportion of visits with symptom related actions (*p* < .0001)
Snyder, C., et al., 2010 [84]	Adults with breast or prostate cancer	Qualitative interviews and Cross-sectional study	Feasibility	None	Not applicable	Not applicable	Not applicable	• Only 2 domains that over 70% of patients reported discussing (pain and information needs), while 9 domains reported by physicians
								• Barriers to using PROs in clinical practice: (1) time constraints, (2) varying relevance of questions, (3) value of the conversational approach, (4) decreased usefulness in established relationships, and (5) respondent burden.
								• Benefits of PROs in clinical practice include (1) identifying problems, (2) serving as a reminder of topics to discuss, and (3) tracking changes over time.
Synder, C. 2014 [85]	Adults with breast or prostate cancer	RCT	Intervention	EORTC QLQ-C30, SCNS-SF34, PROMIS	Clinicians	Problematic scores noted	No instructions given, only usual best practice guidelines	• Patient feedback suggested differences in ratings for included PROs in order QLQ-C30, PROMIS, SCNS-SF34 (P < .05).
								• Clinicians did not prefer one questionnaire over the others.
Taenzer, P. 2000 [86]	Adults with lung cancer	Controlled trial (no randomization)	Intervention	EORTC QLQ-C30, PSQ, PDIS	Nurse, physician	Score information provided	Exit Interview	• No group differences in patient satisfaction
								• More QL issues identified were addressed in the experimental group (p < .001)
								• No statistical differences in charting of issues and action taken.
Takeuchi, E. et al., 2011 [87]	Adults with any cancer type	RCT	Intervention	EORTC QLQ-C30, HADS	Oncologists	Score information provided, graphical display	No instructions given, only usual best practice guidelines	• Significant intervention effect for the discussion of symptoms (p-.008)
								• Discussion of most symptom initiated by patients, no group differences.
								• Severity of symptom associated with clinical discussion. No group differences
Trautmann, F. 2016 [45]	Adults with any cancer type	Cohort study	Implementation	EORTC QLQ-C30, NCCN-DT, BPI	Physician	1 SD above/below mean	Physician-patient consultation using traffic light color-coded scoring	• 79% of patients agreed to participate
								• 67% provided complete PRO information
								• Mean completion time 30 min
								• Rates of approaching patients to participate increased over time
Veilkova, G. 2010 [88]	Adults with any cancer type	RCT	Intervention	EORTC QLQ-C30, HADS	Physician	Not reported	Not reported	• Continuity of care communication rated better in intervention group (o < 03).
								• Stakeholders found PRO to be useful
Wagner, L. 2015 [52]	Adults with ovarian, uterine, or cervical malignancies, non-gynecologic malignancy	Cohort study	Implementation	PROMIS CAT, NCCN- DT, NCCN-Prostate Cancer, PGA	Oncologist, nurse	T scores provided, severity range information	No instructions given, only usual best practice guidelines	• 92% of patients completed at least one assessment
								• Of multiple assessments:
								o 79% Message requests read
								o 37% Assessments started
								o 93% Assessments completed of those started
								• Physical function generated most alerts (4% based on severe problems)
Whittle, A.K., 2016 [89]	Adults with any cancer type: urological, lung,colorectal, breast, and gynecological	Cohort study	Feasibility/ Intervention	CGA GOLD	Clinical team	Score information provided	Questions for patient to ask clinician	• Phase I Observational
								o 42% consent and completion rate
								o 11.7 min mean completion time
								o 86.3% completed CGA-GOLD without assistance.
								o 3.1% missing response rate
								• Phase II Intervention
								o 39% consent and completion rate
								o 89% unchanged decision after comparison of PRO results with clinical notes
Wolfe, J. 2014 [90]	Children with any cancer type	Cluster RCT	Intervention	PediQUEST (PQ): MSAS, PedsQL4.0, sickness	Clinic staff, palliative care service, pain service, patient	Graphical data display	No instructions given, only usual best practice guidelines	• PRO feedback did not have an effect on symptoms and HRQOL in the study, but effect found for children > 8 years surviving > 20 weeks.
								• Report found useful by:
								o 50% of providers
								o 54% of parents
								o 28% of patients
Wolpin, S., 2008 [91]	Adults with any cancer type	RCT	Feasibility/ Intervention	ESRA-C and SDS	Physician	Not reported	Not reported	• Mean completion time 15.20 min 5 out of 6 acceptability questions indicated very high acceptability (mean > 4, on a 1–5 range scale)
Wright, P., et al. 2007 [92]	Adults with any cancer type	Qualitative interviews and Cross-sectional study	Feasibility	SDI, HADS, EORTC QLQ-C30	Social worker investigator	Cut-offs/Thresholds	Under development	• Referral rates 24% for patients above PRO cutoff

^1^ A post-hoc analyses of the data from the same study demonstrated symptom monitoring improves overall survival by 5 months (Basch, 2017). Results were published after the completion of the current review

*Abbreviations*: *AE* = adverse event, *BPI* = Brief Pain Inventory, *CES-D* = Center for Epidemiologic Studies Depression Scale; *CGA GOLD* = comprehensive geriatric assessment screening questionnaire, *CTCAE* = Common Terminology Criteria for Adverse Events, *eCRF* = electronic case report form, *E-MOSAIC* = electronic Monitoring Symptoms and Syndromes Associated with Advanced Cancer; EMR = electronic medical record, *EORTC QLQ-BN20* = European Organisation for Research and Treatment of Cancer Quality of Life Questionnaire Brain Neoplasm, *EORTC QLQ-BR23* = European Organisation for Research and Treatment of Cancer Quality of Life Questionnaire Breast, *EORTC QLQ-C30* = European Organisation for Research and Treatment of Cancer Quality of Life Questionnaire Core 30, *EORTC QLQ-CR38* = European Organisation for Research and Treatment of Cancer Quality of Life Questionnaire Colorectal, *EORTC QLQ-LC13* = European Organisation for Research and Treatment of Cancer Quality of Life Questionnaire Lung Cancer, *EQ-5D* = EuroQol Five Dimensions, *ER* = emergency room, *ESAS* = Edmonton Symptom Assessment Scale; *ESRA-C* = Electronic Self-Report Assessment for Cancer; *FACT-G* = Functional Assessment of Cancer Therapy General, *GP* = general practitioner, *HADS* = Hospital Anxiety and Depression Scale, *HRQoL* = health-related quality of life, *MOS-SS* = Medical Outcomes Study Social Support, *MSAS* = Memorial Symptom Assessment Scale, *NCCN-DT* = National Comprehensive Cancer Network Distress Thermometer, *NCCN-EDT* = National Comprehensive Cancer Network Emotional Distress Thermometer, *PCI* = patients Concerns Inventory, *PDIS* = patient-provider communication, *PEDSQL* = Pediatric Quality of Life Scale, *PGA* = physician’s global assessment, *POMS-17* = Postoperative Morbidity Survey, *PRO* = patient-reported outcome, *PROSQoLI* = Prostate Cancer Specific Quality of Life Instrument, *PSQ-III* = Patient Satisfaction Questionnaire, *PTSD* = post-traumatic stress disorder; *PTSD-CL* = post-traumatic stress disorder civilian, *QLIC-ON* = Quality of Life in Childhood Oncology; *QoL* = quality of life, *RCT* = randomized controlled trial, *REALM-SD* = Rapid Estimate of Adult Literacy in Medicine;m SCNS-SF34 = Supportive Care Needs Survey Short Form, *SDS* = Symptom Distress Scale, *SF-36* = Short-form 36, *SQoL* = subjective quality of life, *STAI* = State Trait Anxiety Inventory, *TAPQoL* = TNO-AZL Preschool Children Quality of Life, *UWQoL* = University of Washington Quality of Life

### Results: Available evidence on reporting of ISOQOL PRO implementation guide categories

Data from all 36 reports of PRO measures used in clinical settings were summarized according to the ISOQOL PRO Implementation Guide Framework [35]. .Publications included research studies (intervention research (*n* = 19, 58%), feasibility research (*n* = 10, 28%), combination intervention and feasibility (*n* = 3, 8%), real-world implementation reports (n = 3, 8%); and an intervention for quality improvement (n = 1, 3%) We initially summarized findings related to design considerations of PRO integration in clinical practice within the specified ISOQOL categories (goals for collecting PROs, assessment details, PRO selection, and mode of administration) followed by evidence in categories related to reporting and use of PRO results (reporting of PRO results, PRO score interpretation, plans for addressing issues identified by the PRO, and evaluation of PRO impact on clinical practice). Table 2 presents a summary of key elements from each paper included in the review.

### Goals for collecting PROs

As part of this synthesis step, we examined the stated clinical-practice goals for the PRO data collection. Planned use of PROs included monitoring symptoms (*n* = 13, 36%), improving quality of care (*n* = 10, 28%), enhancing patient-provider communication (*n* = 9, 25%), and delivering patient-centered care (n = 9, 25%) (Table 3).

**Table 3:** Reported Goals of PRO Inclusion in Clinical Care

PRO Use Goal*	N of Studies	Percent of Studies
Symptom Monitoring	13	36%
Patient-Centered Care	10	28%
Patient-Provider Communication	9	25%
Quality of Care	9	25%
Symptom Screening	5	14%
Symptom Screening/Symptom Monitoring	3	8%
Decision Aid	1	3%

*Abbreviation*: *PRO* = patient-reported outcome

### Patients, setting, and timing of assessments

All studies provided relevant details on the type of patients included, study setting, and timing of assessment (before visit, during visit, after visit, at home). Most studies were conducted with adult populations (94%) in outpatient settings (92%). PROs were administered most often at the clinic immediately before seeing the doctor (36%) or during a visit (33%). The type of cancer patient varied with a majority of studies including three or more cancer types (69%), and only seven studies (19%) with a single cancer type.

### PROs selected for use in the studies

A total of 46 PRO measures were used across the 36 studies; 33 of the PRO measures were rarely used and were included in only one or two studies suggesting wide variability in measures used. Studies predominantly reported measuring symptoms (*n* = 15, 42%) or cancer-specific HRQoL (*n* = 13, 33%) outcomes. The most widely-used measure was the EORTC QLQ-C30 (*n* = 10,28%) followed by the Hamilton Anxiety and Depression Scale (HADS) (*n* = 5, 14%).

### PRO mode of administration

All but one of the studies reported the mode of PRO administration. Electronic administration was the most common mode (*n* = 31, 69%) followed by paper-and-pencil (*n* = 12, 27%). Two studies used interactive voice response (IVR) (*n* = 2, 5%).

### Reporting of PRO results

The summary of information on PRO results indicated that the preferred format of results presentation was an electronic summary report (*n* = 19, 56%) or a printed copy of PRO results (*n* = 7, 19%) while e-mails/telephones (n = 5, 14%) were used less often. Results were most often presented only to the clinical team (*n* = 30, 83%); only three studies (11%) presented the PRO results to both the clinician and the patient, and one study presented results to patients only.

### PRO score interpretation

A large proportion of studies (*n* = 17, 47%) failed to report information on how to interpret PRO scores. Eleven studies (31%) provided PRO scores alone with no interpretation guidance, and six articles (17%) did not report any information on PRO score interpretation. Fifteen studies (42%) provided scores in the form of a graphical display which may aid in score interpretation. About half of the papers reviewed (*n* = 19, 53%) reported PRO scores along with some information on threshold values, cut-off scores, or severity levels. Only three studies (8%) provided reference groups or norms information for the selected PRO.

### Plans for addressing issues identified by the PRO

The majority of studies did not provide any instructions on follow-up steps when PRO scores raised areas of concern. In 13 studies (36%), no instructions were given on next steps or patient-management action items based on PRO results; eight studies (22%) did not include sufficient information on whether PRO results were addressed. The plans for addressing issues identified by the PROs were often related to discussing the identified issues with the provider (*n* = 10, 28%) with single studies also suggesting specialist referrals, reporting adverse events (AEs), and/or providing educational materials to patients.

### Evaluation of PRO impact on clinical practice

Only 19 studies (53%) included results of PRO intervention on patient outcomes. The outcomes for which most studies reported evidence of PRO intervention effect included patient reported symptoms, functioining or quality of life scores (*n* = 13) and patient-provider communication (*n* = 8) (Table 4). Eleven of the 19 studies (58%) reported significant PRO intervention effects for all reported endpoints, and five studies (26%) had mixed results and reported significant PRO intervention effects for some—but not all—of the assessed outcomes. Only three of the 19 studies (16%) reported no intervention effect (Table 2).

**Table 4:** Intervention Study Outcomes and Evidence of PRO Intervention Effect

Type of Outcome*	N^1^ Studies Reporting Intervention Effect	N^1^ Studies Used Outcome	Percent of All Studies Using Outcome
PRO score (symptoms n = 8, functioning n = 1, HRQoL n = 6)	13	16	44%
Discussion of results/patient-centered communication	8	8	22%
Patient satisfaction with treatment	3	4	11%
Chart documentation	3	3	8%
Changes in clinical evaluation or treatment plan	3	3	8%
Number of provider visits	0	2	6%
Emergency-department visits	1	1	3%
One-year survival	1	1	3%

*Abbreviation*: *HRQoL* = health-related quality of life, *PRO* = patient-reported outcome

^1^A total of 19 intervention studies reporting results on study outcomes were included in the review. Multiple outcomes assessed across studies

### Results: Real-world implementation of PRO measures with oncology patients

Our review identified only three reports of real-world implementation of PRO measures in clinical practice which we defined as the ongoing administration of a standardized PRO and related clinical actions to manage patient care in routine clinical practice beyond the scope of a specific research study.

The first study [43] used retrospective chart review to investigate the relationship between standardized symptom screening and clinical actions to manage symptoms using the Edmonton Symptom Assessment Scale (ESAS) [44]. The ESAS was included in routine clinic visits though self-reporting via an electronic touch-screen kiosk. The ESAS measures the severity (scale of 0–10; 0 = none, 10 = worst) of nine common cancer physical and psychological symptoms (pain, shortness of breath, nausea, anxiety, depression, tiredness, drowsiness, appetite, and well-being). ESAS symptoms were categorized into four severity categories: none (0 score), mild (1–3 score), moderate (4–6 score), and severe (7–10 score) where scores of > 4 indicate clinically-significant symptom issues. Symptom-related actions included relevant drugs being prescribed, medication dosage titration, or a test, treatment, or referral being made. Pain and shortness of breath were documented in 52% and 30% of charts; a related action occurred in 17% and 4% of charts, respectively. However, the frequency of relevant clinical actions was not proportionate to the documented symptom severity [43].

Trautmann and colleagues [45] described the development, implementation, completeness, and first results of an electronic, real-time assessment program for the collection of PROs in a tertiary referral cancer center in Germany. The EORTC QLQ-C30 [46], National Comprehensive Cancer Network Distress Thermometer (DT) [47], and the Hornheider Screening Instrument (HIS) of need for psycho-oncological support [48] were measured. Nutritional status was assessed using the Short-Form Mini Nutritional Assessment (MNA) [49], and pain was assessed using the Brief Pain Inventory (BPI) [50, 51]. A traffic-light system was applied for visualized score interpretation using published cutoff values or means/standard deviations (SDs). A green light indicated scores below a critical clinical importance thresholds based on means and standard deviations of reference population of cancer patients thereby indicating no need for clinical action; a red light indicated scores above a critical cut-off that indicated need for further action. Overall, 67% of patients provided complete information on 12 PROs. Rates of approach and participation varied between the different departments with the highest completion rates in patients presenting for oncological surgical consultation. The number of patients approached to complete PROs increased from 17% to 56% over three months. The percentage of patients completing the PROs increased from 70% to 92% over three months. The majority of patients (62%) reported a score of five or higher on the NCCN Distress scale indicating moderate to high burden; 53% of the patients had a score of four or higher on the HSI indicating a need for psycho-oncological support. Very few participants reported on pain outcomes as EORTC QLQ-C30 pain intensity, and impairment scores were only documented in patients reporting moderate to severe pain. Findings revealed that physician usage of PRO during the clinical consultation was limited. Limiting factors reported by physicians were the lack of knowledge of the PRO reporting system and perceived irrelevancy of some of the assessed PRO data. Rates of clinical action were not reported in the study.

The authors acknowledged a number of obstacles in the study—even though there was an increased number of patients recruited, the usage of PROs in the patient-physician interaction was limited due to physician turnover and lack of completion time provided to patient prior to consultation. The authors concluded that PRO assessments should be more carefully selected to be more clearly of benefit to the health care provider and patient. Additionally, sustaining the implementation and interpretation of PROs should be constantly reinforced with clinicians [45].

Wagner and colleagues [52] assessed cancer-related symptoms with electronic health record (EHR) integration to communicate assessment results to clinical teams in real time. PROMIS computer adaptive tests (CATs) use a computer algorithm developed with item response theory to administer the items. The psychosocial assessment was adapted from the National Comprehensive Cancer Network Distress Thermometer and Problem Checklist [53]. Over the course of three years, 636 patients completed a total of 1493 assessments with 636 patients completing the assessment at least once (301 twice, 184 three times, and 129 four times). Most patients (90.1%) completed the assessment at home rather than at the clinic (9.3%). Severe PROMIS symptom scores (≥70 or 75 depending on symptom) triggered a message to the oncology team. PROMIS T-score clinical severity thresholds (normal, mild, moderate, or severe) have been previously determined with a standard setting exercise that converged clinician expert ratings and patient self-reported severity scores [54]. Overall, one-third of the patients reported current psychosocial health needs. The authors consider that this study demonstrates that precise measurement of symptoms can be implemented while maintaining the brevity required for clinical implementation. EEHR integration also facilitated automated triage for psychosocial and supportive care [52].

## Discussion

### Evidence on reporting of ISOQOL PRO implementation guide categories

In order to be able to follow the ISOQOL PRO Implementation Guide, researchers need to have a body of evidence to guide choices on recommended categories.The first aim of our review was to examine existing information on recommended implementation based on published information from oncology clinical settings. While no studies in our review directly referenced the ISOQOL PRO Implementation Guide [35], publications on PRO use in oncology clinical practices were well aligned in their reporting of most recommended implementation elements. However, a gap exists in the description of PRO interpretation guidelines and attendant patient-management recommendations necessary to improve PRO outcomes.

Most studies adequately described the planned goal for PRO data collection, study setting, and selection of PRO and mode of administration. Electronic administration, which allows for flexible integration of PROs in clinical care, was used by the majority of studies, but most were not formally integrated into the electronic health record. In addition, formal integration into the EHR may require intensive resources and stakeholder buy-in that have been lacking perhaps due to limited evidence of the improvement in patient outcomes or lack of financial alignment (or incentives).

While the EORTC QLQ-C30 was the most commonly-used measure across studies, a wide variety of PRO measures was reported suggesting there is little consensus on core domains or the “best” PRO to use or consideration whether a measure is developed for clinical trial or clinical practice use. Such variability may be an implementation barrier for PROs in everyday oncology practice—the burden to individual organizations associated with selecting PRO measures, developing assessment guidelines for the selected PROs, and interpreting PRO results may discourage adoption in routine clinical care.

The main gap in evidence identified by this review was the sparsity of interpretation guidelines for PRO results provided to care providers. While most of the reviewed studies provided PRO scores, fewer added interpretation guidelines to these scores or provided follow-up instructions or procedures in case a problem was identified by the PRO. This is an important gap. Without clarity on the meaning, significance, and interpretation of collected PRO data, how can clinical actions be effected to result in improved health care processes and outcomes?

### Real-world implementation of PRO measures with oncology patients

Based on the review of the published literature, the use of PRO measures in routine cancer clinical practice outside the context of feasibility or research intervention studies is seldom reported. Only three reports of routine implementation of PROs in clinical settings were identified by the current review and provided limited information for our first key aim The multi-stage process that is required for developing, introducing, testing, integrating, and monitoring PROs in EHR systems has been achieved in only a few US medical centers [55]. Numerous barriers to implementation have been discussed in the literature including: (1) perception among clinicians that PRO completion consumes valuable time during the patient visit; (2) EHR systems have limited abiity to deliver PROs in user-friendly formats for patients; and (3) clinical ecosystem workflow demands challenge full implementation and integration into clinical practice [56].

While our review suggests that PRO implementation in real-world settings outside of research context is scarce, it is possible that PRO implementation in real-world oncology clinical practice may be underreported in the research literature. Implementation efforts may be viewed more as quality-improvement efforts that are building on existing evidence but not always viewed as generating evidence warranting dissemination [23]. Therefore, it is plausible that PRO implementation may be more widespread than indicated by existing peer-reviewed publications. In a recent article, Basch and colleagues [55] noted that a handful of institutions have successfully integrated systematic PRO collection into routine clinical practice; however, no published data have been generated from these institutions related to real-world PRO implementation in oncology clinical care.

### Comparison to other systematic literature reviews

Some of our findings are consistent with the results from earlier literature reviews evaluating different aspects of PROs in the context of oncology care (Table 5). We confirmed earlier findings that there is evidence for the effectiveness of PROs on improving provider-patient communication and increased discussion of mental health issues [23, 31–34]. The EORTC QLQ-C30 was also found to be the most commonly-evaluated PRO in oncology clinical practice settings [32]. The wide variability of PRO measures used has also been noted [55] and continues to be a challenge as confirmed by our findings. Several of the earlier reviews also pointed out the need for increased attention in providing guidance for PRO implementation in oncology clinical practice [33, 34, 57]. Since the completion of our review window, several additional reviews have appeared that focus on some aspect of PRO use in oncology clinical care such as mechanisms through which PROs facilitate increase in patient-physician communication [58] and use of PROs specifically in treating lung cancer [59]. The unique contributions of the current review remains, as no other review focused on separately examining PRO real-world implementation reports or used the ISOQOL PRO Implementation Guide as the framework in analyzing the identified articles. The use of this framework has allowed us to identify specific gaps in the PRO implementation cycle that need to be addressed to encourage use in clinical practice―mainly, the insufficient focus on developing and providing clear PRO score interpretation guidelines and patient-management action plans related to PRO results.

**Table 5:** Summary of Earlier Relevant SLR of Implementation of PRO in Cancer Clinical Care

Review Reference	Review goal	Databases + Search strategy	# References Screened	# Articles reviewed	Timeframe	Major Conclusions
Howell et al. 2015 [32]	To identify PROMs used in routine cancer clinical practice, their impact on patient, provider, and system outcomes, and the implementation factors influencing uptake.	Ovid Medline CINAHL PsycINFO Grey Literature	3297	30	2003–2013	The EORTC QLQ30 was the most commonly used PRO Use of PROMs for screening for emotional distress, unmet supportive care needs, or social difficulties Wide variety of PROMs were used with little standardization across studies PROMs implementation improves communication about symptoms and QoL More attention needs to be paid to complexity of implementation and interpretation of PROMS
King et al. 2016 [33]	To examine the use and impact of using quality of life measures on health care of cancer patients within a clinical setting, particularly those with brain cancer.	PubMed, EMBASE, Cochrane (SR & Trials), Web of Science [SCI]) Grey literature	18,483	19	2000–2015	QoL data may improve patient–physician communication, increase discussion of emotional functioning in particular. Scarcity of data on actions/medical decisions.
Chen et al. 2013 [31]	To provide a comprehensive review update including all relevant quantitative studies investigating the effectiveness of routine PRO collection in cancer patients.	NR Two-step search strategy building on existing reviews	1182	27	2000–2011	Strong evidence: PROs enhances patient-provider communication, improves patient satisfaction. Moderate evidence: PRO improves monitoring of treatment response and the detection of unrecognized problems. Weak Evidence: Changes to patient management, improved health outcomes No evidence: changes to patient health behavior, quality improvement, increased transparency, accountability, public reporting and better health care system performance
Jensen et al. 2013 [93]	To identify existing PRO systems and their administration of PRO assessments, integration of information into the clinic workflow and EHR systems, and the reporting of PRO information.	PubMed MEDLINE	190 plus conference abstracts and gray literature	33 ePRO systems reviewed	Not specified, conferences 2009–2011	Identified systems were generally developed to improve symptom management, identify psychosocial problems, and facilitate patient-provider communication. Data on actual impact was not part of review scope
Antunes et al. 2014 [57]	To systematically identify facilitators and barriers to the implementation of patient-reported outcome measures in different palliative care settings for routine practice	Medline, PsycINFO, Cumulative Index to Nursing Allied Health Literature, Embase British Nursing Index	3863	31	1985–2011	There is a need for guidance on implementing PROMs in palliative care clinical practice.
Alsaleh 2013 [23]	To review the scientific evidence behind recommending the use of QoL scales routinely in outpatient evaluation.	Medline, Embase, PsycINFO	486	6	1990–2012	Evidence for the use of QoL scales in daily clinical practice is limited. Some weak evidence suggesting that this might improve communication between patients and health caregivers. No good evidence that routine administration of QoL questionnaires improve patient’s QoL or changes management. The overall impression is that routine administration of questionnaires in medical oncology outpatient clinics is currently hardly justified.
Luckett et al. 2009 [94]	To identify future strategies for PRO interventions to impact patient outcomes in cancer clinics	MEDLINE PsycINFO References from earlier review included	576	6	2006–2008	More trials are urgently needed to build a satisfactory evidence base for the routine clinical use of patient-reported data in oncology. Evidence for improvement in patient outcomes as result of PROM use has been limited
Kotronoulas et al. 2014 [34]	Is inclusion of PROM in routine clinical practice associated with improvements in patient outcomes, processes of care, and health service outcomes during active anticancer treatment	Medline, EMBASE, CINAHL, PsycINFO, PBSC	5015	26	Database inception −2012	Use of PROMs increases the frequency of discussion of patient outcomes. Some support for positive association between use of PROSM and improved symptom control, increased supportive care measures, and patient satisfaction. Need for additional effort to ensure patient adherence and clear system guidelines to guide clinicians response. More research needed to support cost-benefit.
Yang et al. 2017 [58]	To identify mechanisms through which PROs facilitate patient-clinician communication in the adult oncology population.	MEDLINE, EMBASE, CINAHL, PsycINFO, Cab Direct, CDSR	610	43	Prior to 2016	PROs facilitate patient-clinician communication through various mechanisms that could perhaps contribute to improvements in symptom management and survival. The impact of PROs on clinical outcomes, however, remains poorly studied.
Bouazzaa et al. 2017 [59]	To analyze the use of PROMs in the treatment of lung cancer with the aim of improving the quality of care. The secondary objective is to evaluate PROMS currently being used in the care of lung cancer.	PubMed, Web of Science and Google Scholar	1118	51	2010–2016	There has yet to be a study on the routine implementation of lung cancer specific PROMs, but PROMs have a promising role.

*Abbreviations*: *EHR* = electronic health record, *ePRO* = electronic patient-reported outcome, *EORTC QLQ-C30* = European Organisation for Research and Treatment of Cancer Quality-of-Life Questionnaire-Core 30, *PRO* = patient-reported outcome, *PROM* = patient-reported outcome measure, *QoL* = quality of life

### Strengths and limitations

The strengths of this review include the compliance with PRISMA guidelines, development of a comprehensive two-step search strategy, and review of results in the context of a framework built on the ISOQOL PRO Implementation Guide [35]. The results of the review help further the conversation on PRO implementation in oncology clinical practice by identifying gaps in guidance on interpretation of PRO results and action-oriented patient management based on PRO results. The review also has some limitations including the relatively small number of databases included in the review.

## Conclusion

The existing evidence of PRO implementation in real-world clinical care in the published literature is very limited. It is unclear whether implementation efforts are not being studied, not being reported in peer-reviewed journals, simply being published in the grey literature, or not taking place at all.

While publication on PRO real-world implementation is uncommon, a good number of publications on PRO feasibility and/or PRO use in research in oncology clinical care exists as evidenced by our work and earlier literature reviews. This paper also aimed to organize findings of published studies in a framework informed by the ISOQOL PRO Implementation Guide [35]. Results suggested that, with the notable exception of PRO score interpretation and action strategies for PRO-identified problems, most studies report information suggested by the ISOQOL PRO Implementation Guide.

Based on the findings from our review, we offer two insights to help enable more widespread PRO implementation in routine clinical practice. First, adequate interpretation guidelines are needed for PRO results to be acted upon in clinical practice. Second, exploration should be conducted into how to best address issues raised by PRO results—particularly when the identified needs of patients extend beyond the expertise or training found in a routine oncology clinical practice such as depression or lack of social support. In the absence of available information on these key elements, implementation of PROs in clinical practice is unlikely to bridge the gap between perceived usefulness by researchers and routine uptake in oncology practice by clinicians.

## Abbreviations

ASCO: American Society of Clinical Oncology
BPI: Brief Pain Inventory
DT: Distress Thermometer
EHR: Electronic health record
EORTC QLQ-C30: European Organisation for Research and Treatment of Cancer Quality-of-Life Questionnaire-Core 30
ESAS: Edmonton Symptom Assessment Scale
HIS: Hornheider Screening Instrument
HRQoL: Health-related quality of life
ISOQOL: International Society for Quality of Life Research
MNA: Mini Nutritional Assessment
PRISMA: Preferred Reporting Items for Systematic Reviews and Meta-Analyses
PRO: Patient-reported outcome
SD: Standard deviation
SLR: Systematic literature review
US: United States
